# Case Report: Early T-cell precursor acute lymphoblastic leukemia with aberrant CD19 expression and an *NPM1* mutation

**DOI:** 10.3389/fonc.2026.1890324

**Published:** 2026-07-22

**Authors:** Yan Lu, Yiran Chen, Qiaohong Zhang, Gongqiang Wu

**Affiliations:** 1 Clinical Laboratory, Affiliated Dongyang Hospital of Wenzhou Medical University, Dongyang, China; 2 School of Medicine, Hangzhou City University, Hangzhou, China; 3 The Department of Hematology, Affiliated Dongyang Hospital of Wenzhou Medical University, Dongyang, China

**Keywords:** ETP−ALL, flow cytometry, FLT3−ITD, next−generation sequencing, NPM1 mutation

## Abstract

**Introduction:**

Early T-cell precursor acute lymphoblastic leukemia (ETP-ALL) is a high-risk subtype of T-cell acute lymphoblastic leukemia (T-ALL). Aberrant CD19 expression in ETP−ALL may lead to misdiagnosis of mixed-phenotype acute leukemia. Notably, *nucleophosmin 1 (NPM1)* mutations, an established molecular hallmark of acute myeloid leukemia (AML), are extremely rare in T−ALL.

**Case report:**

We present the case of a 29−year−old male with progressive right retroauricular and cervical lymphadenopathies complicated by severe febrile neutropenia and polymicrobial sepsis. Peripheral blood testing revealed 80% blasts. Bone marrow flow cytometry demonstrated blasts strongly positive for cytoplasmic CD3 and CD7, with aberrant CD19 expression and co−expression of CD34, CD33, and dim cytoplasmic CD79a. The blasts showed dim CD5 expression but were negative for CD1a, CD8, CD10, CD22, and myeloperoxidase, consistent with an ETP-ALL immunophenotype. Next−generation sequencing identified *an NPM1* frameshift mutation (p.W288Cfs*12, Type A) and an internal tandem duplication of the *Fms−like tyrosine kinase 3* gene *(FLT3*−ITD*)*, both classic AML−associated mutations. After management of severe sepsis, the patient received three cycles of VHAG (venetoclax, homoharringtonine, cytarabine, and granulocyte colony-stimulating factor)−based chemotherapy followed by haploidentical allogeneic hematopoietic stem cell transplantation (allo-HSCT). He remained in sustained complete remission at the 7-month follow−up after allo-HSCT.

**Conclusion:**

To our knowledge, this case represents the first reported ETP-ALL with an *NPM1* frameshift mutation (p.W288Cfs*12, Type A), which also exhibited aberrant CD19 expression, highlighting the diagnostic challenges posed by unusual immunophenotypic and genomic profiles.

## 1 Introduction

Early T-cell precursor acute lymphoblastic leukemia (ETP-ALL) accounts for 10–15% of adult T-cell acute lymphoblastic leukemia (T-ALL), and is characterized by an arrest of T-cell differentiation with stem cell or myeloid marker expression (1). ETP-ALL is associated with poor prognosis, chemotherapy resistance, a high frequency of myeloid-related gene mutations, and a high risk of severe infectious complications at initial presentation (2).

Aberrant expression of B-lineage markers, particularly cluster of differentiation 19 (CD19), in T-ALL represents a major diagnostic pitfall (3). Strict application of the World Health Organization (WHO) lineage assignment criteria is critical to avoid misdiagnosis as mixed−phenotype acute leukemia (MPAL) (4).

*Nucleophosmin 1 (NPM1)* frameshift mutations (p.W288Cfs*12, Type A) are present in approximately 30–35% of acute myeloid leukemia (AML) cases and are highly AML−defining, with only rare occurrence in other myeloid neoplasms and acute lymphoblastic leukemia (5–7).

The uniqueness of this case lies in its concurrent presentation of two features, namely an *NPM1* frameshift mutation (p.W288Cfs*12, Type A), which, to our knowledge, has not been previously reported in ETP-ALL, and aberrant CD19 expression, a well-recognized diagnostic pitfall that may lead to misclassification. Additionally, the patient experienced severe polymicrobial sepsis at diagnosis, which required urgent anti-infective management prior to leukemia-directed therapy. This case expands the molecular and immunophenotypic spectrum of ETP-ALL and provides valuable information for the diagnosis and management of infectious complications as well as application of targeted therapies.

## 2 Case description

### 2.1 Clinical presentation

In early May 2025, a 29−year−old male presented with a 3−day history of progressive right retroauricular, cervical, and mandibular swelling and distending pain. He reported an occipital rash, oropharyngeal discomfort, sore throat, and mild dry cough without headache, bleeding diathesis, or other systemic symptoms. The patient’s medical history was unremarkable.

During the outpatient visit, the patient presented with a fever of 40.2 °C. Laboratory testing showed leukopenia (white blood cell count 0.74 × 10^9^/L, absolute neutrophil count 0.06 × 10^9^/L) with 80% circulating blasts and markedly elevated high−sensitivity C−reactive protein levels (306.42 mg/L). Cervical ultrasonography revealed multiple enlarged lymph nodes in the right retroauricular, cervical, and submandibular regions. The patient was admitted to our hospital on May 7, 2025, for further evaluation and management.

### 2.2 Diagnostic assessment

The bone marrow aspirate smear was hypocellular, with decreased myeloid lineage cells. It was dominated by marked lymphoblast proliferation, accounting for approximately 74% of all nucleated cells, a finding that is morphologically consistent with ALL.

**Figure 1:**
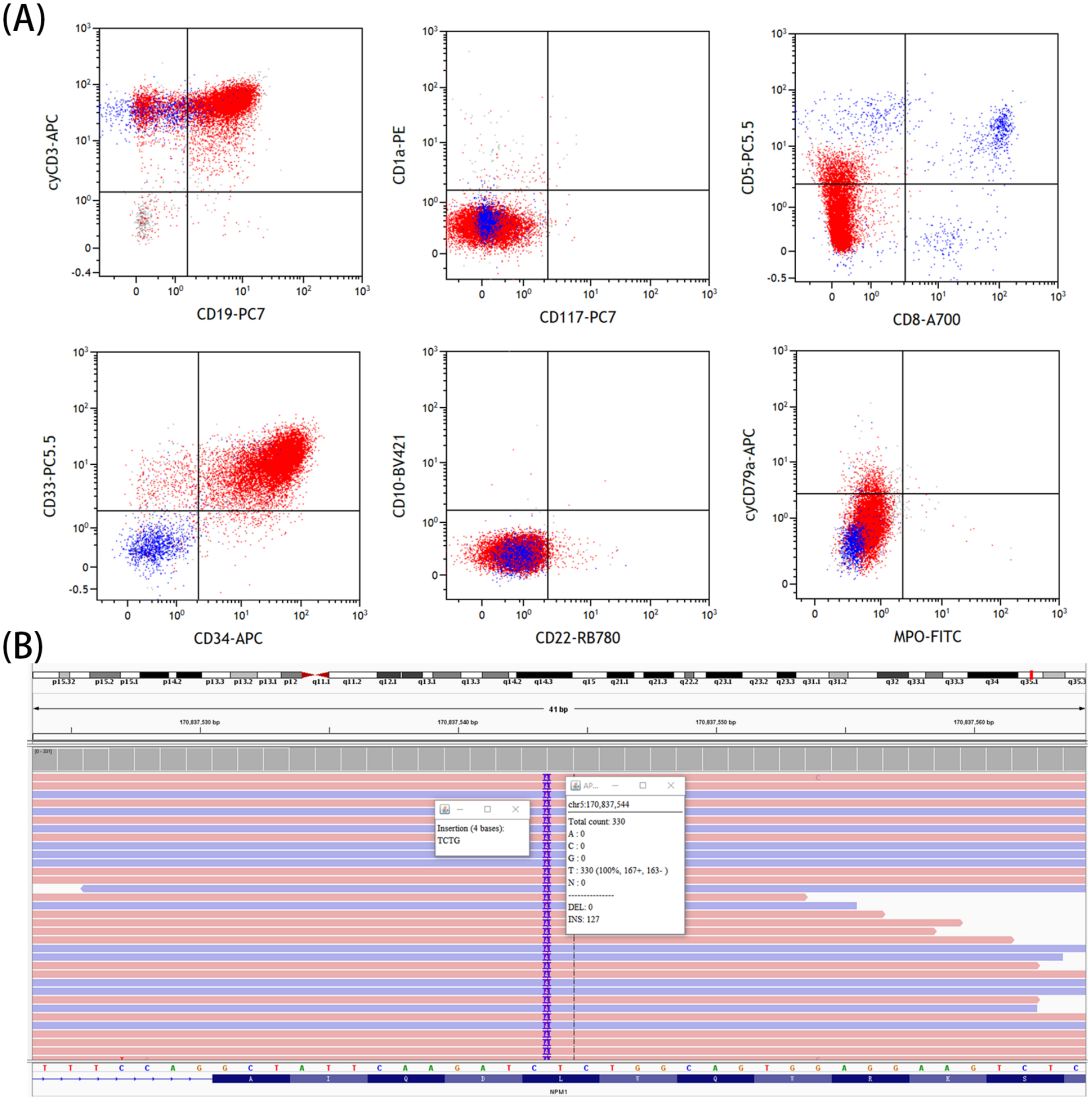
**(A)** Multiparametric flow cytometry of bone marrow aspirate. Red dots indicate leukemic blasts, and blue dots denote normal mature T lymphocytes. Blasts show an immunophenotype consistent with early T−cell precursor acute lymphoblastic leukemia (ETP−ALL): positive for cytoplasmic CD3 (cyCD3), with aberrant expression of CD19, CD34, and CD33; negative for CD1a, CD8, CD117, MPO, CD10, and CD22; and dim expression of CD5 and cytoplasmic CD79a (cyCD79a). **(B)** Next-generation sequencing electropherogram confirms the presence of the *NPM1* mutation (p.W288Cfs*12).

Multiparameter flow cytometry analysis identified an abnormal blast population constituting 78.60% of marrow-nucleated cells. Using a CD45/SSC gating strategy, the blasts were analyzed for antigen expression intensity. The median fluorescence intensity (MFI) ratio was calculated for each marker relative to an appropriate internal negative control population. Mature T lymphocytes served as the internal negative control for non-T-lineage markers, while B lymphocytes served as the internal negative control for T-lineage markers.​ Based on our laboratory’s established criteria, an MFI ratio of <2.0 was defined as negative, 2.0–4.0 as dim positive, and >4.0 as bright positive. Borderline samples with MFI ratios ranging from 1.8–2.2 or 3.8–4.2 were comprehensively interpreted combined with multi-marker co-expression profiles, and retesting with alternative antibody fluorescence channels was performed when necessary to prevent misclassification. As shown in **Figure 1A**, the blasts were strongly positive for cytoplasmic CD3 (cyCD3) and CD7 (all MFI ratios >10.0) and aberrantly co-expressed CD19 (MFI ratio 9.1), along with CD34, CD33, and dim cytoplasmic CD79a (cyCD79a; MFI ratio 2.3). They lacked CD1a, CD8, CD10, CD22, and myeloperoxidase (MPO), with dim CD5 expression (MFI ratio 2.7). Additional negative markers included CD3, CD2, CD4, CD117, CD11b, CD14, CD64, CD300e, TdT, and cyCD22 (all MFI ratios <1.5).

Next-generation sequencing identified a frameshift mutation in *NPM1* (p.W288Cfs*12, Type A) at a variant allele frequency (VAF) of 42.9% (**Figure 1B**) and an *FLT3* internal tandem duplication (*FLT3*-ITD) at a VAF of 28.9%. Additional copy-number alterations (CNAs) included *ETV6* deletion, *RB1* deletion, 12p−, and 13q−. No pathogenic fusion genes or germline-predisposing mutations were identified.

Chromosomal analysis showed a normal karyotype (46, XY) with limited evaluable metaphase material due to insufficient cell proliferation.

Differential diagnoses included typical T-ALL, B/T MPAL, AML with aberrant T-lineage markers (especially the *NPM1* mutation), and T-lymphoblastic lymphoma. The differential diagnoses​ and reasons for exclusion are summarized in **Table 1**. According to the WHO 5^th^ Classification of Haematolymphoid Neoplasms (WHO-HAEM5) criteria for lineage assignment in acute leukemia (4), the diagnosis of ETP−ALL was established.

**Table 1:** Key differential diagnoses and resolution for the present case.

Diagnostic entity	Key immunophenotypic & molecular features	Basis for exclusion in this case
Typical T-ALL	Definitive T-lineage commitment (cyCD3^+^/sCD3^+^), CD7^+^; typically CD1a^+^ or CD4/CD8 co-expression, bright CD5 expression; absent stem-cell/myeloid markers.	Excluded. The blasts showed CD1a−, CD8−, dim CD5 expression, and aberrant expression of stem-cell/myeloid markers CD34 and CD33. These features are atypical for T-ALL but are pathognomonic for ETP-ALL.
B/T MPAL	Requires definitive evidence of dual-lineage commitment per WHO-HAEM5 criteria: 1. B-lineage: CD19^+^ plus strong expression of at least one of CD10, CD22, or cyCD79a 2. T-lineage: strong cyCD3^+^/sCD3^+^	Excluded. While blasts showed strong CD19 and cyCD3 expression, they lacked a second definitive B-lineage marker (CD10^−^, CD22^−^, cyCD79a only weakly positive), failing to meet the WHO criteria for B-lineage assignment. Dual-lineage commitment was not confirmed.
AML with Aberrant T-Lineage Markers	Definitive myeloid commitment: MPO+ (or monocytic marker expression), CD13+, CD33+, CD117+; *NPM1* mutation is a defining molecular feature of AML.	Excluded. Blasts were MPO-negative and negative for monocytic markers (CD11b, CD14, CD64, CD300e), with strong cyCD3 expression, definitively establishing T-lineage commitment. The presence of *NPM1* and *FLT3*-ITD mutations (typical of AML) does not override the confirmed T-lineage immunophenotype.
T-Lymphoblastic Lymphoma	Identical immunophenotype to T-ALL/ETP-ALL; distinguished by disease extent: limited to nodal/extranodal tissue with <25% bone marrow blast involvement.	Excluded. The patient had 78.60% bone marrow blast infiltration, which fulfills the diagnostic criteria for acute lymphoblastic leukemia rather than lymphoma.

T-ALL, T-cell acute lymphoblastic leukemia; ETP-ALL, early T-cell precursor acute lymphoblastic leukemia; MPAL, mixed-phenotype acute leukemia; AML, acute myeloid leukemia; cyCD3, cytoplasmic CD3; sCD3, surface CD3; cyCD79a, cytoplasmic CD79a; MPO, myeloperoxidase; WHO-HAEM5, World Health Organization 5th Classification of Haematolymphoid Neoplasms; *FLT3*-ITD, *FLT3* internal tandem duplication.

### 2.3 Therapeutic intervention

Following admission in May 2025, the patient was managed in a laminar airflow bed for severe neutropenia and a febrile state. Initial management focused on a life-threatening polymicrobial infection, identified by blood next-generation sequencing to include *Staphylococcus aureus*, *Escherichia coli*, and respiratory syncytial viruses, among others. Antimicrobial therapy with imipenem and vancomycin was initiated, but was complicated by drug-induced hallucinations, leading to a switch to meropenem. Diagnostic flow cytometry (May 9th) confirmed ETP-ALL with aberrant CD19 expression. After a brief self-directed transfer to an external hospital for continued infection control, the patient returned to our center.

**Figure 2:**
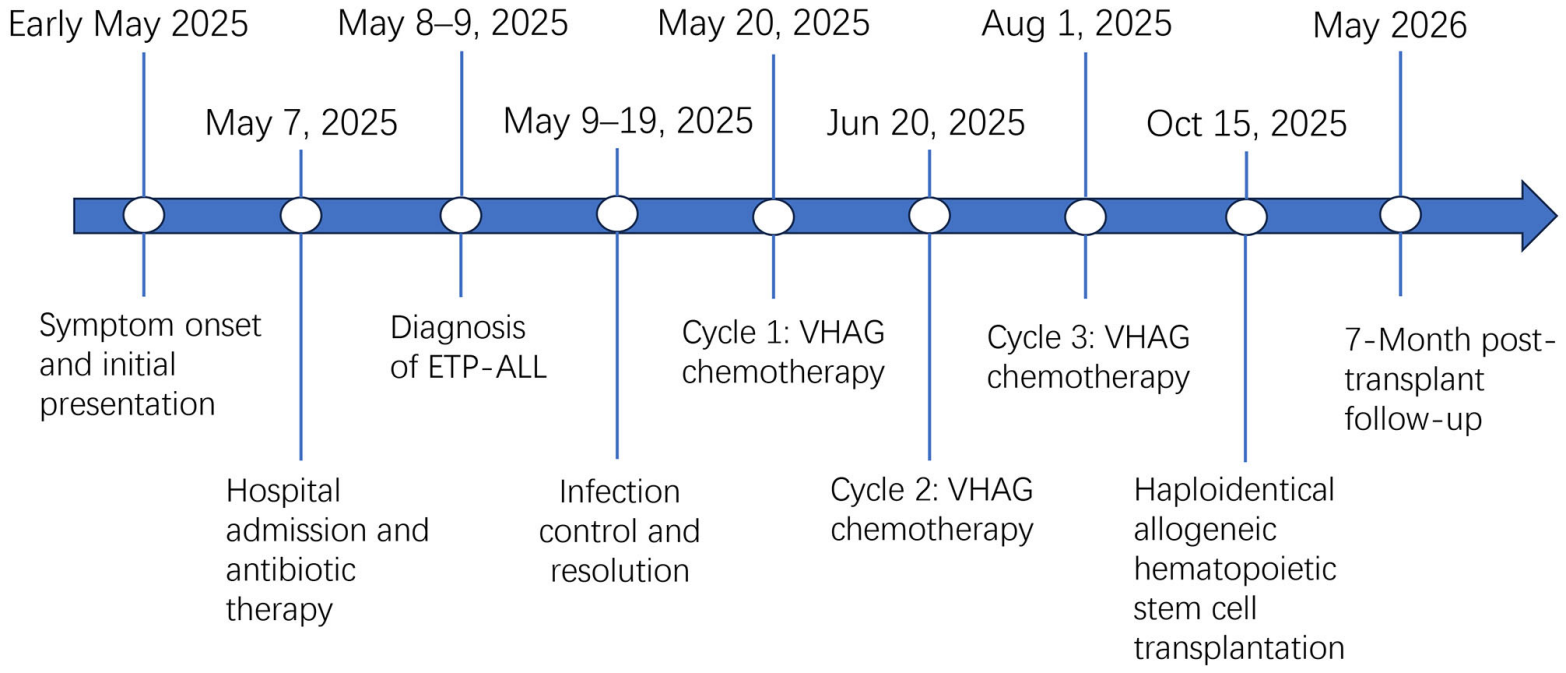
Timeline of clinical presentation, diagnosis, treatment, and follow-up.

Upon resolution of infectious complications by mid-May, definitive leukemia therapy was initiated. The patient received three cycles of the VHAG regimen (venetoclax, homoharringtonine, cytarabine, and granulocyte colony-stimulating factor) commencing on May 20, June 20, and August 1, 2025. This was followed by a haploidentical allogeneic hematopoietic stem cell transplantation (allo-HSCT) on October 15, 2025. The clinical timeline is shown in **Figure 2**. The myeloablative conditioning regimen consisted of cytarabine, busulfan, semustine, cyclophosphamide, and anti-thymocyte globulin. Post-transplant graft-versus-host disease (GVHD) prophylaxis included cyclosporine, mycophenolate mofetil (discontinued after 1 month), and prednisone. The immunosuppressive regimen was gradually tapered during follow-up, and the current maintenance regimen was adjusted to oral cyclosporine 25 mg twice daily combined with prednisone 5 mg once daily.

### 2.4 Follow-up and outcomes

The patient underwent serial monitoring for minimal residual disease (MRD) in the bone marrow on June 3, June 18, July 26, and September 5, 2025. None of the four flow cytometric assessments showed evidence of abnormal early precursor T lymphoblasts. On July 26, 2025, personalized molecular MRD testing targeting the patient’s specific mutation profile was performed, and both the *NPM1* mutation and *FLT3*−ITD were undetectable.

The patient underwent haploidentical allo-HSCT on October 15, 2025. Post-transplant chimerism was assessed by short tandem repeat polymerase chain reaction at months 1, 3, and 6, with all assessments demonstrating complete donor chimerism (>95% donor cells in both whole blood and CD3+ fraction). Bone marrow MRD by flow cytometry was performed monthly for the first 6 months post-transplant, and all evaluations remained negative.

Mild cutaneous GVHD (National Institutes of Health grade 1–2) developed around December 10, 2025, presenting as limited skin involvement. The condition was managed with the existing immunosuppressive regimen (cyclosporine and prednisone), with gradual dose tapering. Currently, the patient is maintained on cyclosporine 25 mg BID and prednisone 5 mg QD, with only minimal residual skin rash.

At the most recent follow-up in May 2026 (approximately 7 months post-transplantation), the patient remained in sustained complete remission with excellent performance status. Apart from manageable skin GVHD, the patient exhibited no clinical or laboratory evidence of infectious complications or leukemic relapse. Long-term post-transplant surveillance will include chimerism analysis every 6 months. Bone marrow MRD by flow cytometry will be performed every 2 months until 1 year post-transplant, and then every 3–6 months thereafter. Long-term monitoring for late effects of transplantation, including endocrine function, cardiac health, and secondary malignancies, will be performed annually.

## 3 Discussion

ETP-ALL is a biologically and clinically distinct subtype of T-ALL. In this case, the patient exhibited a classic ETP-ALL immunophenotype accompanied by two highly unusual features: aberrant CD19 expression and an *NPM1* frameshift mutation (p.W288Cfs*12, Type A), both of which pose substantial diagnostic challenges.

Aberrant CD19 expression in T-ALL may lead to misdiagnosis as MPAL or B-cell ALL (B-ALL). Per the WHO-HAEM5 criteria, B-lineage commitment requires CD19 expression together with strong expression of at least one of the following: CD10, CD22, or cyCD79a. In our patient, CD19 was aberrantly positive, CD10 and CD22 were negative, and cyCD79a was weakly expressed. Furthermore, cyCD79a is expressed in up to 10% of T-ALL cases and cannot be reliably used to define B-lineage commitment once the T-lineage is confirmed (8). These findings emphasize that diagnostic errors can be avoided only by strict adherence to standardized immunophenotypic criteria.

The identification of an *NPM1* mutation in this case is particularly noteworthy, as *NPM1* mutations (p.W288Cfs*12, Type A) are considered highly characteristic of AML yet are extremely rare in lymphoid leukemia. Therefore, to support the novelty of this finding, we performed a comprehensive literature search across PubMed, Embase, and Web of Science using the combined search terms “ETP-ALL” OR “Early T-cell precursor acute lymphoblastic leukemia” AND “*NPM1* mutation, ” including all publications up to May 2026. No previously reported case of ETP-ALL harboring an *NPM1* frameshift mutation was identified. We acknowledge that genomic alterations involving the *NPM1* locus, particularly copy−number loss associated with 5q deletion, have been described in ETP−ALL (9). However, this is distinct from the canonical *NPM1* gene frameshift mutation (type A) identified here via next-generation sequencing. To our knowledge, this is the first genomically confirmed case of an *NPM1* frameshift mutation in ETP-ALL. Although myeloid-associated gene mutations, including *FLT3*, *NRAS/KRAS*, *DNMT3A*, *IDH1*, and *IDH2*, are commonly reported in ETP-ALL (10), specific mutations have been linked to prognosis. For instance, *IDH2* is associated with higher relapse risk and inferior survival in T-ALL (11). As a classic AML-defining mutation, the *NPM1* frameshift mutation has not been reported in ETP-ALL previously, and its prognostic role in this setting remains unclear. The co-occurrence of *NPM1* and *FLT3*-ITD mutations in this patient complicates prognostic interpretation, as these two alterations carry opposing prognostic implications in AML. At the same time, their coexistence reinforces the molecular overlap between early T-cell neoplasms and myeloid malignancies, supporting the concept that ETP-ALL arises from early hematopoietic stem cells with multilineage differentiation potential, thereby blurring the traditional boundaries between myeloid and lymphoid lineages.

In addition to gene mutations, the present case harbored recurrent CNAs including *ETV6* deletion, *RB1* deletion, 12p-, and 13q-. These CNAs are biologically significant in T-ALL/ETP-ALL, as *ETV6* inactivation disrupts hematopoietic transcription (12), and 13q14 deletion involving *RB1* and *LPAR6* impairs cell-cycle control and has been linked to ex vivo chemoresistance (13). While *ETV6* alterations, as part of broader co-lesion patterns, are enriched among early immature and near-ETP T-ALL subtypes (14), the strongest mechanistic evidence associating *ETV6* with high-risk phenotypes and chemotherapy resistance in T-ALL is limited to individual case reports (15, 16). Rigorous multivariate survival analyses exclusively dedicated to T-ALL cohorts remain sparse to date. The coexistence of these CNAs with *NPM1* and *FLT3*-ITD mutations indicates a highly complex genomic background. Given that such complexity is associated with adverse outcomes in other acute leukemias (17, 18), this profile supports the use of intensive therapy and close post-treatment MRD monitoring for risk-adapted management.

The treatment strategy for ETP-ALL remains poorly standardized, and there is no universally accepted first-line regimen for this high-risk entity. Homoharringtonine, a natural plant alkaloid, exerts anti-leukemic effects by inhibiting cellular protein synthesis and triggering apoptosis of leukemic blasts; it also possesses potent cytotoxic activity against both lymphoid and myeloid leukemic cells. Given that this patient harbored two AML-associated driver mutations (*NPM1* frameshift mutation and *FLT3*-ITD) and aberrantly expressed myeloid markers including CD33 and CD34, we opted for the VHAG regimen, which can target both lymphoid and myeloid malignant clones. In addition, the VHAG regimen was selected over classic T-ALL regimens such as Hyper-CVAD (hyperfractionated cyclophosphamide, vincristine, doxorubicin, and dexamethasone) due to its lower intensity and more favorable tolerability profile, which were particularly advantageous given the patient’s critical condition with severe polymicrobial sepsis at presentation. During treatment, serial detection of immunophenotypes and molecular mutations was conducted to monitor potential lineage shift under chemotherapy pressure. The dosage and administration cycle of VHAG were optimized based on evidence from domestic clinical trials involving patients with high-risk acute leukemia (19). The potential role of FLT3 inhibitors, including sorafenib, midostaurin, and gilteritinib, was carefully considered given the presence of *FLT3*-ITD. However, because the patient achieved a deep molecular response with undetectable MRD after VHAG chemotherapy, additional FLT3 inhibitor therapy was deemed unnecessary. Currently, the safety and efficacy of combining FLT3 inhibitors with VHAG or using these agents as post-transplant maintenance therapy for ETP-ALL remains unvalidated. Further clinical investigations are warranted to explore FLT3-targeted therapy in patients with ETP-ALL, particularly those with refractory disease or persistent MRD positivity.

From a clinical perspective, the patient’s initial presentation was complicated by life-threatening severe neutropenic sepsis with a polymicrobial infection that required prompt and targeted antimicrobial therapy before leukemia-directed treatment could be initiated. Successful control of infection is critical for subsequent chemotherapy and transplantation. The patient’s favorable outcome following VHAG-based chemotherapy and definitive haploidentical transplantation highlights the feasibility of curative-intent therapy even for high-risk ETP-ALL with complex molecular features.

The primary strength of this case is its exemplary model of integrated diagnostics​ and molecularly informed therapy​ for highly challenging leukemia. The main limitation is that, although the case described genotype-phenotype differences, the potential biological mechanisms were not investigated. Nevertheless, this case provides valuable lessons on the diagnostic pitfalls and the multidisciplinary management of rare ETP-ALL phenotypes.

This case demonstrates that ETP-ALL may exhibit aberrant CD19 expression, which should not lead to its misclassification as MPAL or B-ALL. *NPM1* and *FLT3*-ITD mutations, which are typically associated with AML, can also occur in ETP-ALL supporting the molecular overlap between early T-lineage and myeloid neoplasms. A comprehensive diagnostic evaluation combining morphology, flow cytometry, and next-generation sequencing is essential for accurate diagnosis, appropriate risk stratification, and personalized clinical management.

## 4 Patient perspective

The patient reported significant distress from pain, fever, and lymph node swelling at the initial presentation. The patient tolerated antimicrobial therapy, chemotherapy, and allo-HSCT with manageable side effects. He expressed satisfaction with the diagnostic clarity, treatment efficacy, and sustained complete remission at the 1-year follow-up. The patient remained compliant with regular follow-ups and was satisfied with his current quality of life.

## Data Availability

The original contributions presented in the study are included in the article/supplementary material, further inquiries can be directed to the corresponding author/s.
